# Accommodating Dynamic Oceanographic Processes and Pelagic Biodiversity in Marine Conservation Planning

**DOI:** 10.1371/journal.pone.0016552

**Published:** 2011-02-02

**Authors:** Hedley S. Grantham, Edward T. Game, Amanda T. Lombard, Alistair J. Hobday, Anthony J. Richardson, Lynnath E. Beckley, Robert L. Pressey, Jenny A. Huggett, Janet C. Coetzee, Carl D. van der Lingen, Samantha L. Petersen, Dagmar Merkle, Hugh P. Possingham

**Affiliations:** 1 The Ecology Centre and Centre for Applied Environmental Decision Analysis, University of Queensland, St. Lucia, Australia; 2 The Nature Conservancy, West End, Australia; 3 Botany Department, Nelson Mandela Metropolitan University, George, South Africa; 4 Climate Adaptation Flagship, CSIRO Marine and Atmospheric Research, Hobart, Australia; 5 Climate Adaptation Flagship, CSIRO Marine and Atmospheric Research, Cleveland, Australia; 6 Centre for Applications in Natural Resource Mathematics (CARM), School of Mathematics and Physics, University of Queensland, St. Lucia, Australia; 7 School of Environmental Science, Murdoch University, Murdoch, Australia; 8 Marine and Coastal Management, Department of Environmental Affairs and Tourism, Rogge Bay, South Africa; 9 Marine Research Institute, University of Cape Town, Rondebosch, South Africa; 10 WWF Responsible Fisheries Programme, WWF South Africa, Cape Town, South Africa; National Institute of Water & Atmospheric Research, New Zealand

## Abstract

Pelagic ecosystems support a significant and vital component of the ocean's productivity and biodiversity. They are also heavily exploited and, as a result, are the focus of numerous spatial planning initiatives. Over the past decade, there has been increasing enthusiasm for protected areas as a tool for pelagic conservation, however, few have been implemented. Here we demonstrate an approach to plan protected areas that address the physical and biological dynamics typical of the pelagic realm. Specifically, we provide an example of an approach to planning protected areas that integrates pelagic and benthic conservation in the southern Benguela and Agulhas Bank ecosystems off South Africa. Our aim was to represent species of importance to fisheries and species of conservation concern within protected areas. In addition to representation, we ensured that protected areas were designed to consider pelagic dynamics, characterized from time-series data on key oceanographic processes, together with data on the abundance of small pelagic fishes. We found that, to have the highest likelihood of reaching conservation targets, protected area selection should be based on time-specific data rather than data averaged across time. More generally, we argue that innovative methods are needed to conserve ephemeral and dynamic pelagic biodiversity.

## Introduction

There has been a substantial decline in the diversity and abundance of pelagic species worldwide owing to pressures from overfishing, pollution, climate change, eutrophication, and invasive species [1], [2], [3]. In particular, overfishing has resulted in the collapse of numerous fisheries, the decline of many species, and in some instances, changes in the structure and functioning of entire ecosystems [4], [5], [6], [7]. This has been, at least in part, due to management objectives that focus on maximizing the catch of target species, while overlooking interactions within ecosystems [8]. In response, a large body of theory has been developed on Ecosystem-Based Management (EBM), where ecosystems are managed holistically and management actions planned across all user sectors [2], [9]. In principle there has been considerable support for this approach, but implementation has been problematic, mostly due to the complexities of balancing multiple and often conflicting objectives [10].

One management approach that has become increasingly popular for supporting EBM is the establishment of area-based management, such as protected areas, where management regulates human activities within designated boundaries [11], [12]. Protected areas have been applied predominantly in coastal and benthic environments [13], but more recently they have been suggested for the pelagic realm [14], [15]. Pelagic protected areas are likely to be particularly effective where species occur predictably at some point in time and management can reflect this predictability. For example, sea turtles often occur regularly along frontal systems in offshore areas [16], [17]. In Hawaii, daily information predicting loggerhead turtle habitat based on oceanographic characteristics is used to help guide fisheries management [18]. Pelagic protected areas are also expected to perform well for species whose feeding or breeding aggregations that are spatially restricted [19]. Nonetheless, the occurrence of many pelagic species can vary dramatically in both space and time, because of variability in physical and ecological processes that determine their distribution and abundance [14], [20]. Because of this dynamic variability, the utility of pelagic protected areas to conserve pelagic biodiversity is contentious [15], [21], [22].

The science of conservation planning emphasizes the use of specific conservation objectives, and the application of decision support tools to help identify where, how and when these objectives can most efficiently be achieved [23]. Applying conservation planning methods requires an understanding of the spatial configuration of different habitats and species and the location of components of an ecosystem that require the most urgent action. Ideally, there will also be some understanding of the likely ecological, social, economic, cultural and political consequences of implementing conservation actions. Conservation planning methods for the representation of habitat types in systems of benthic and coastal protected areas are well developed e.g. [24]. Although important challenges remain for including dynamic processes in conservation planning, new methods are emerging. These include using time-series data on oceanographic features and species occurrences to identify important areas for management that are predictable e.g. [16] and to identify important areas where management might be required to vary in space and time in response to system dynamics e.g. [25], [26].

### Designing a system of pelagic protected areas in the southern Benguela and Agulhas Bank ecosystems

**Figure 1:**
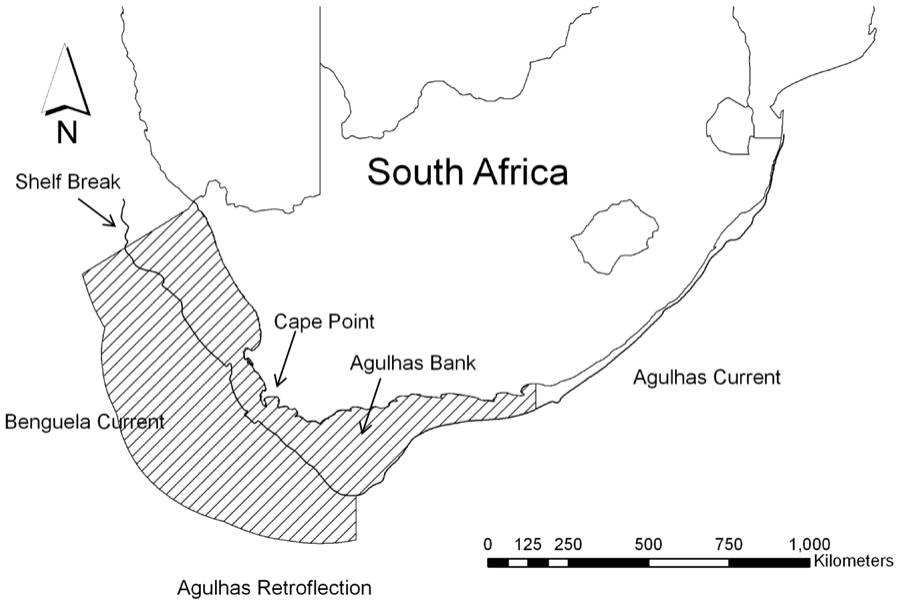
The study region comprising the South African section of the Benguela and the Agulhas Bank ecosystems (hatched area). The outer boundary of the study region is the South African exclusive economic zone.

The southern Benguela and Agulhas Bank ecosystems off the west and south coasts of South Africa (Fig 1) comprise a globally significant marine region renowned for its prodigious fisheries and unique biodiversity [27], [28], [29]. It forms part of the Benguela Current Large Marine Ecosystem, one of the four major eastern boundary current upwelling zones of the world [30] and the Agulhas Current Large Marine Ecosystem, one of the largest western boundary currents in the world. The inshore ecosystem is characterized as a “wasp-waist” diversity pattern, comprising high species diversity at low and high trophic levels, but lower diversity at the mid-trophic level [31]. The main mid-trophic species are clupeids: sardines (*Sardinops sagax*), anchovies (*Engraulis encrasicolus*), and round herring (*Etrumeus whiteheadii*) [32], [33]. Zooplankton constitutes a large part of the diet of these fishes; sardines feed on both phyto- and zooplankton, anchovies are predominantly zooplanktivorous, and round herring feed only on zooplankton [34]. Further, the spawning of many fish species coincides with the maximum food availability of zooplankton (copepod) for their larvae [35], [36]. Ecological dynamics of this region are complex and the movement patterns of many species are not well understood [27].

Fisheries have been identified as one of the major threats to biodiversity objectives in the southern Benguela and Agulhas Bank ecosystems [37], [38]. Accordingly, complementing the management of South Africa's fisheries with a network of protected areas has been identified in a recent conservation assessment as a major management goal to assist with the sustainable management of marine resources [39]. There is evidence that the establishment of a system of no-take protected areas might provide insurance against the decline of some species due to overfishing, provide baseline monitoring areas free from fishing, and supplement the production of fishery species in surrounding fished areas [40], [41], although there is some debate surrounding some of these claims [42].

Our aim here was to demonstrate a decision-support system to assist in the systematic design of a network of pelagic protected areas representing key fisheries species and species of conservation concern in the southern Benguela and Agulhas Bank ecosystems, South Africa. Using the conservation planning software Marxan [43],we developed a flexible planning approach that accounts for the dynamics of pelagic species and habitats by using data on major oceanographic processes and the abundance of small pelagic fishes.

The overall aim of the decision support system was to identify areas that achieved quantitative targets for conservation features, while minimizing the cost to the South Africa fishing industry. We define a conservation feature as an element of conservation interest considered in the design of a protected area network. To map the distribution of conservation features, we used a combination of oceanographic and species data (Table 1). To predict areas important for the conservation of pelagic species, such as areas of high primary productivity, we used four types of data related to two spatially-fixed and four spatially-variable (flexible, below) oceanographic processes. The fixed processes were areas of elevated productivity caused by two types of geological features, the shelf break and seamounts. Both feature types are important drivers of elevated productivity throughout the water column [14], [44]. A flexible process is defined as an oceanographic or biological feature that is not fixed in space [45]. The four important flexible processes used were coastal upwelling, offshore eddies and filaments, areas of retention, and primary consumers.

**Table 1:** Features used in the design of pelagic protected areas in the southern Benguela and Agulhas Bank ecosystems.

Type of feature	Data	Number of targets used in analysis	Period of data (if applicable)
*Oceanographic process*			
Elevated productivity caused by shelf	Polygon	1	
Elevated productivity caused by seamounts	Polygon	4	
Coastal upwelling	Monthly composite image of chlorophyll *a* (0.0833° resolution grid)	84	2000–2006
Freq. of up- and down-welling eddies and filaments	Summary of 10 years of sea surface height images (0.33° resolution grid)	2	1993–2003
Retention areas	Output of a Lagrangian particle-tracking model	1	
*Biological processes*			
Copepods	Interpolated annual surveys of copepod biomass (5km2 resolution grid)	14	1988–2001
Annual sardine density	Interpolated bi-annual surveys (0.0045° resolution grid)	24	1987–2007
Annual anchovy density	Interpolated bi-annual surveys (0.0045° resolution grid)	24	1987–2007
*Species data*			
Fisheries species	Density distribution maps (fisheries and research surveys 0.6° resolution)	8	
Coastal birds	Polygon of foraging distances from colonies	5	
By-catch species	Catch rates (1998–2005) (1° resolution grid)	7	

We included in our analysis several pelagic species that are heavily harvested. These include sardines, anchovies and round herring caught in the small pelagic purse-seine fishery, horse mackerel (*Trachurus trachurus capensis*) and chub mackerel (*Scomber japonicus*) caught predominantly in the mid-water trawl fishery, tunas (*Thunnus* spp.) and swordfish (*Xiphias gladius*) caught in the pelagic longline and tuna pole fisheries, and inshore, squid (*Loligo vulgaris reynaudii*) and numerous teleost species, including predatory fish such as snoek (*Thyrsites atun*) and geelbek (*Atractoscion aequidens*) caught using traditional hook and line. Many of these species are relatively common and are likely to have important functional roles in the ecosystem [46], [47], [48].

Protected areas might also conserve non-targeted species in the southern Benguela and Agulhas Bank. Fisheries are likely to have contributed to a decline in coastal seabird populations, some of which are endemic to the region [19]. Coastal seabirds are typically central-place foragers, and feed primarily on small pelagic fishes close to nests while nesting. While there is uncertainty as to the causes of decline in these species, it has most likely resulted from a combination of a shift in the distribution of prey away from foraging areas, disturbance by fishing boats, feeding on low quality fisheries waste, predation by Cape Fur Seals, feral cats, kelp gull attacks and competition with the purse-seine fishery for prey within foraging areas [49], [50], [51]. It has been suggested that increased protection of their prey within the foraging areas of seabirds, particularly during the breeding season, might help mitigate this decline particularly for African penguins (*Spheniscus demersus*), bank cormorants (*Phalacrocorax lucidus*), Cape cormorants (*Phalacrocorax capensis*), Cape gannets (*Morus capensis*), and roseate terns (*Sterna dougallii* [19], [51], [52]. Although there is debate regarding this issue [53].

Many offshore pelagic species are threatened as by-catch from fishing [54]. In offshore areas, eddies move through the southern part of the system from the Agulhas retroflection, producing favourable habitat for swordfish and tuna, both targeted by the longline fishery [55]. This habitat is also preferred by several species of oceanic seabirds, turtles and sharks that are all in decline owing to by-catch from fisheries here and throughout the world [54], [56], [57]. We included in our analysis the most frequently caught by-catch species in the South African pelagic longline fishery which include three seabirds (black-browed albatross *Thalassarche melanophrys*, shy albatross *T. cauta/steadi*, and white-chinned petrel *Procellaria aequinoctialis*), two turtles (leatherback *Dermochelys coriacea* and loggerhead *Caretta caretta*), and two sharks (short-finned mako *Isurus oxyrinchus* and blue *Prionace glauca*). Protected areas could potentially help reduce the decline of many of these species and consequently we included these in our analysis.

Our analysis consisted of four scenarios. For the first scenario, we designed a protected area network that captured the spatial and temporal dynamics of pelagic species and habitats in the region. For this scenario we set conservation targets for different time periods for pelagic features to be captured within the protected area network (see Table 1). For example, for sardines we set a target for its annual abundance for each year we had data (1987–2007) and assumed that targeting its abundance of previous years will capture the spatio-temporal dynamics of future years. We compared this approach to three other scenarios. For scenario two, the data for pelagic features were based on average values over the time period considered for each dataset. This is a commonly used approach in conservation planning e.g. [45]. For the third scenario, we varied a parameter in the analysis to increase the size of areas selected offshore past the shelf as the spatial and temporal dynamics are greater offshore compared to inshore. As pelagic conservation planning is likely to be applied in combination with benthic conservation planning, the fourth scenario combined benthic and pelagic protected area design. We demonstrate how benthic features, such as a benthic habitat map, can be incorporated into this approach. This study provides a general approach for delivering systematic conservation planning in pelagic ecosystems that could be used for other regions.

## Results

**Figure 2:**
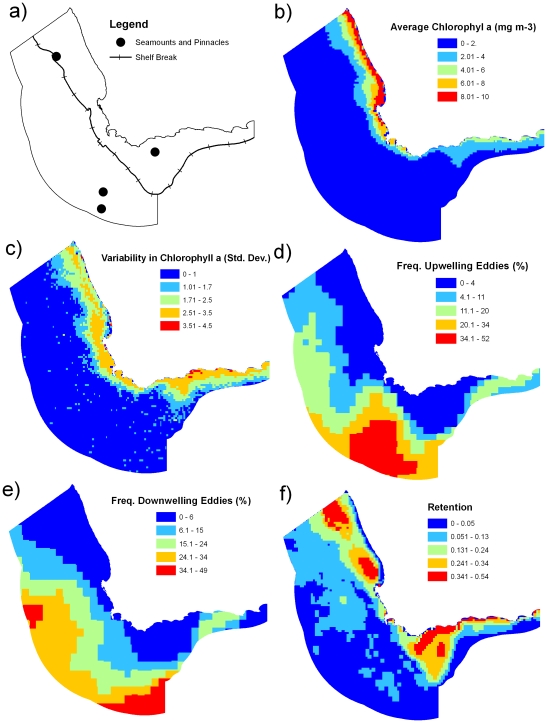
Oceanographic features used in the design of pelagic protected areas. (a) seamounts and shelf break, (b & c) chlorophyll *a*, (d) frequency of upwelling eddies and filaments, (e) frequency of downwelling eddies and filaments, (f) retention.

**Figure 3 pone-0016552-g003:**
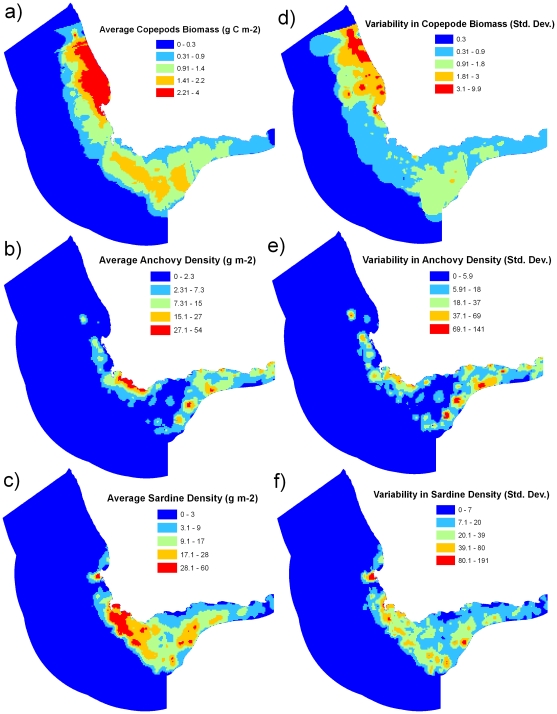
Biological processes used in the design of pelagic protected areas. (a) and (d) copepod biomass, (b) and (e) anchovy densities, (c) and (f), sardine densities.

We found oceanographic “processes” (table 1) were variable in intensity and location throughout the study region (Fig 2a–f). Chlorophyll *a*, was, on average, highest on the west coast (Fig 2b), but was quite variable within this area (Fig 2c). Both upwelling and downwelling eddies and filaments occurred in the southern part of the study region, while downwelling eddies and filaments also occurred on the western boundary (Fig 2d–e). Retention was highest on the west and south-west coasts (Fig 2f). Copepod biomass was, on average, highest on the west coast (Fig 3a). Copepods also had a high average biomass on the Agulhas Bank (Fig 3a), similar to the pattern observed in sardines and anchovy densities (Fig 3b,c), although average anchovy density was not evenly spread across the Agulhas Bank. Copepods, sardines and anchovies all had high variability throughout their distributions (Fig 3d–f).

**Figure 4:**
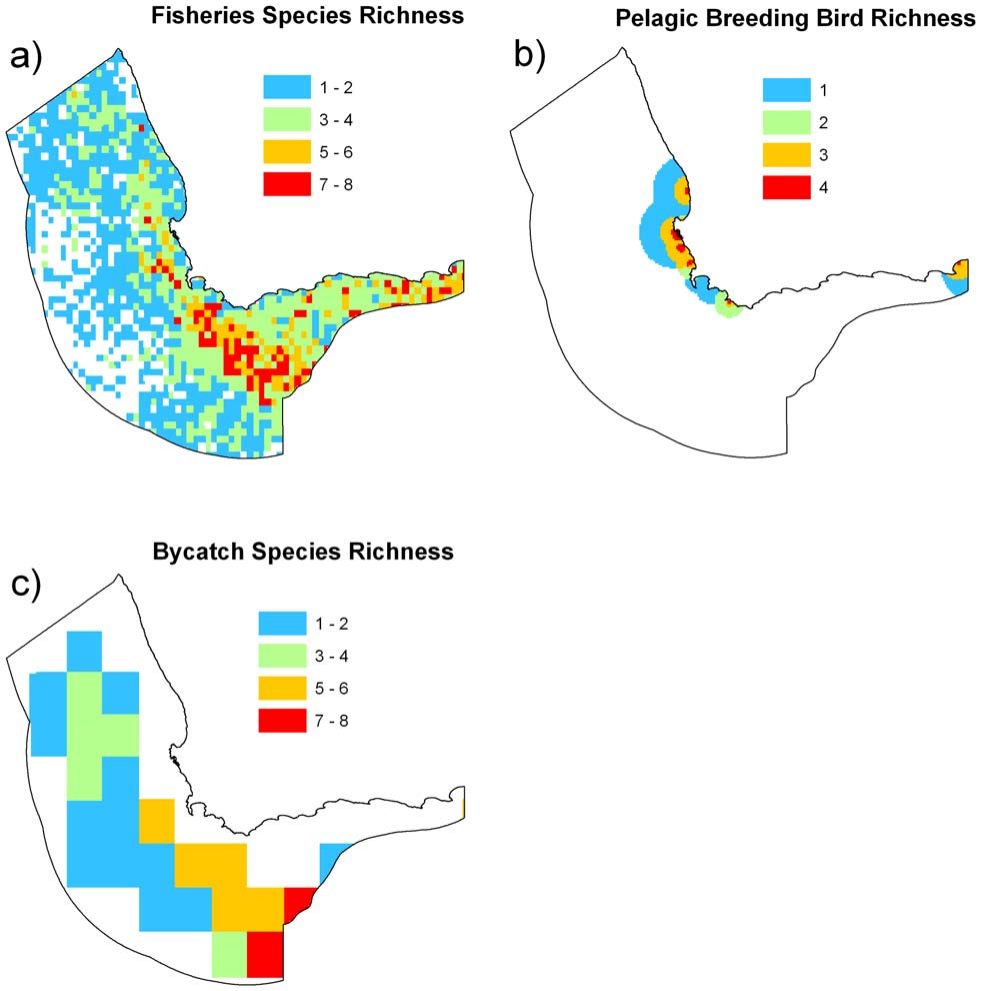
Species richness of key fisheries species and species of conservation concern. (a) eight fisheries species based on density distributions, (b) five pelagic breeding bird species based on breeding foraging range, (c) seven species caught as by-catch (three seabirds, two turtles and two sharks) based on catch rates. Density distribution and catch rate values were converted into presence-absence data with any value >0 recorded as present.

For the shelf region (Fig 2a), we found that the area surrounding Cape Point and the western part of the Agulhas Bank had highest richness of fisheries species, coastal bird foraging areas and, to a lesser extent, by-catch species (Fig 4a–c). There was also high species richness of fisheries species in the eastern coastal area of the study region. High species richness surrounding Cape Point overlapped with high chlorophyll *a*, moderate copepod biomass and some high-density areas of anchovies and sardines (Fig 2–4). High species richness on the western Agulhas Bank overlaps with high retention, moderate copepod biomass, and areas of high densities of anchovies and sardines. In offshore areas, the southern region had the highest richness of species caught as by-catch (Fig 4c), which overlapped with areas of frequent eddies and filaments (Fig 2d–e).

Each time Marxan is run, it is likely to produce a slightly different final solution because the number of potential solutions makes it nearly impossible to identify a single global optimum. Marxan was run 1000 times to produce two outputs, a “best solution”, which is the run that best achieved the objectives, and “selection frequency” a measurement of how frequently an area/planning unit was selected across all 1000 runs. The selection frequency better indicates the importance of an area for achieving objectives and the best solution provides an indication of an individual solution.

**Figure 5:**
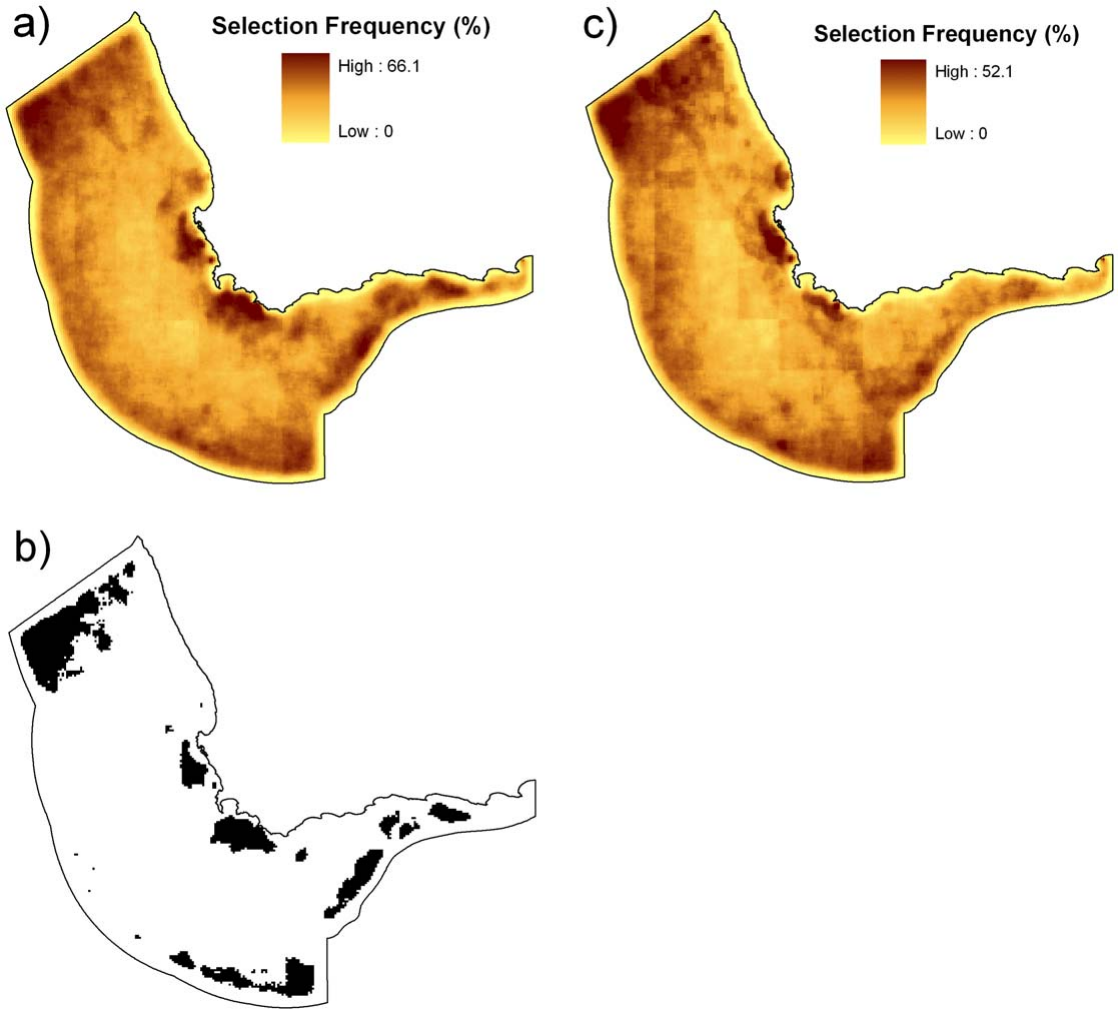
Selection frequencies for two scenarios. The selection frequency is the number of times a particular planning unit was selected across 1000 runs and is used as an indication of conservation importance. For each planning unit (candidate area for selection) the value represents the percentage of 1000 repeat runs in which it was selected. Both results were based on the same targets except that (a) had targets representing different time periods for chlorophyll *a* (monthly), copepods (yearly), anchovies (yearly) and sardines (yearly), (b) had targets based on the averaged values, for the full periods of data availability, for chorophyll *a*, copepods, anchovies and sardines and (c) planning units that have a selection frequency value one standard deviation higher from the mean selection frequency with targets representing different time periods.

Many possible alternate protected area networks were able to meet our objectives so that on average most planning units appeared in some of the final solutions (Fig 5a). The mean and standard deviation for planning unit selection frequency were 25% and 9% respectively. The most frequently selected planning unit appeared in only 66% of solutions, a good indication that there is high spatial flexibility meeting protected area objectives. Locations with high selection frequency (measured as one deviation from the mean) were located in the north-western, southern and eastern boundaries, in addition to two areas around Cape Point (Fig 5b).

The presence of a large number of conservation features surrounding Cape Point and the western Agulhas Bank is likely to explain the higher selection frequency within these areas (Fig 5a). We did find that the eastern and northwestern parts of our study region also had higher selection frequencies. This area overlapped with the highest frequency of both upwelling and downwelling eddies and filaments (Fig 3d–e) and probably explains why higher selection frequencies resulted (Fig 5a). Selection frequency is also probably influenced by these areas being relatively cheap (according to our simple cost function), while still contributing to targets for several conservation features. Other offshore areas, mainly along the north-western and southern boundary, also had higher selection frequencies, because of the high cost effectiveness of achieving targets in these locations.

**Figure 6:**
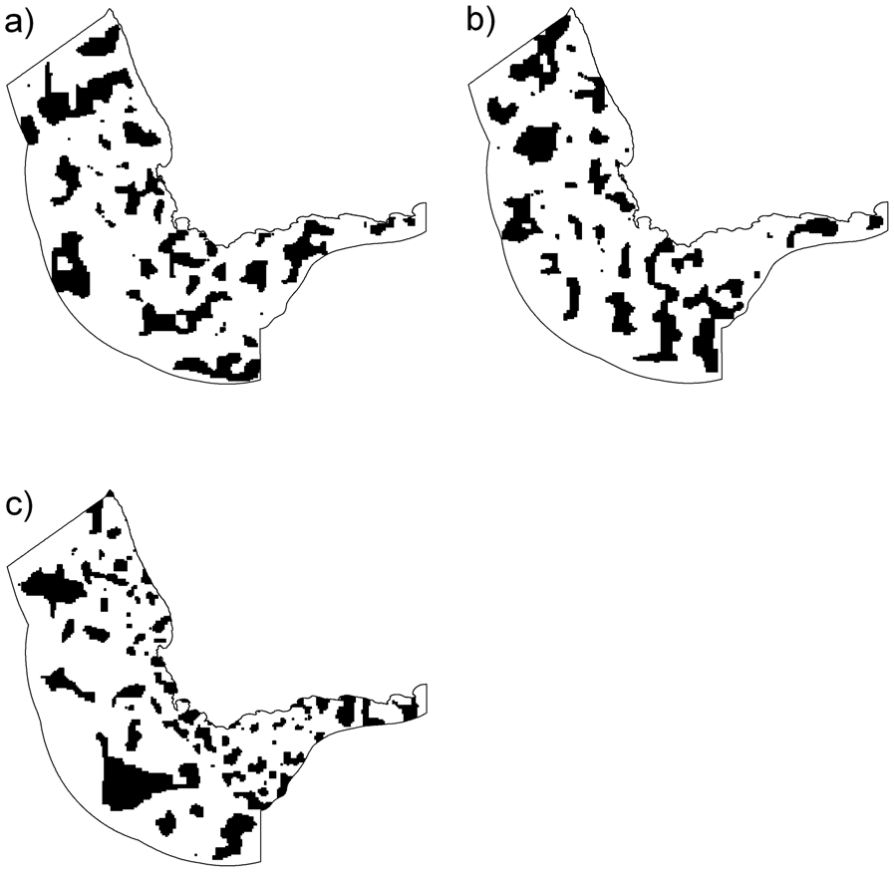
The most efficient protected area solutions for three scenarios. (a) and (b) had targets of the same size but representing features with data aggregated over different time periods. For (a), values for chlorophyll *a* were monthly, copepods yearly, anchovies yearly and sardines yearly. For (b), values for chorophyll *a*, copepods, anchovies and sardines were averaged over the entire periods of data availability. Boundary lengths, which help to determine the compactness of the area configurations, were the same in parts (a) and (b). For (c), boundary lengths between planning units were longer offshore than inshore to produce solutions with more compactness offshore.

We compared the results of scenario one with scenario two that used the average values for the time periods covered by data for chlorophyll *a*, copepods, anchovies and sardines, there were high correlation of selection frequencies (Spearman's rank correlation of 0.88, p<0.0001) (Fig 5a&c). When comparing the best solutions of these two scenarios (Fig 6a–b), we found that achievement of targets for averaged data required fewer planning units (n = 4764) than for features split by time periods (n = 4988). The proportion of total cost based on summing the cost metric values across all planning units was also similar between the averaged data best solution (18.46%) and features split by time period best solution (20.88%).

**Figure 7:**
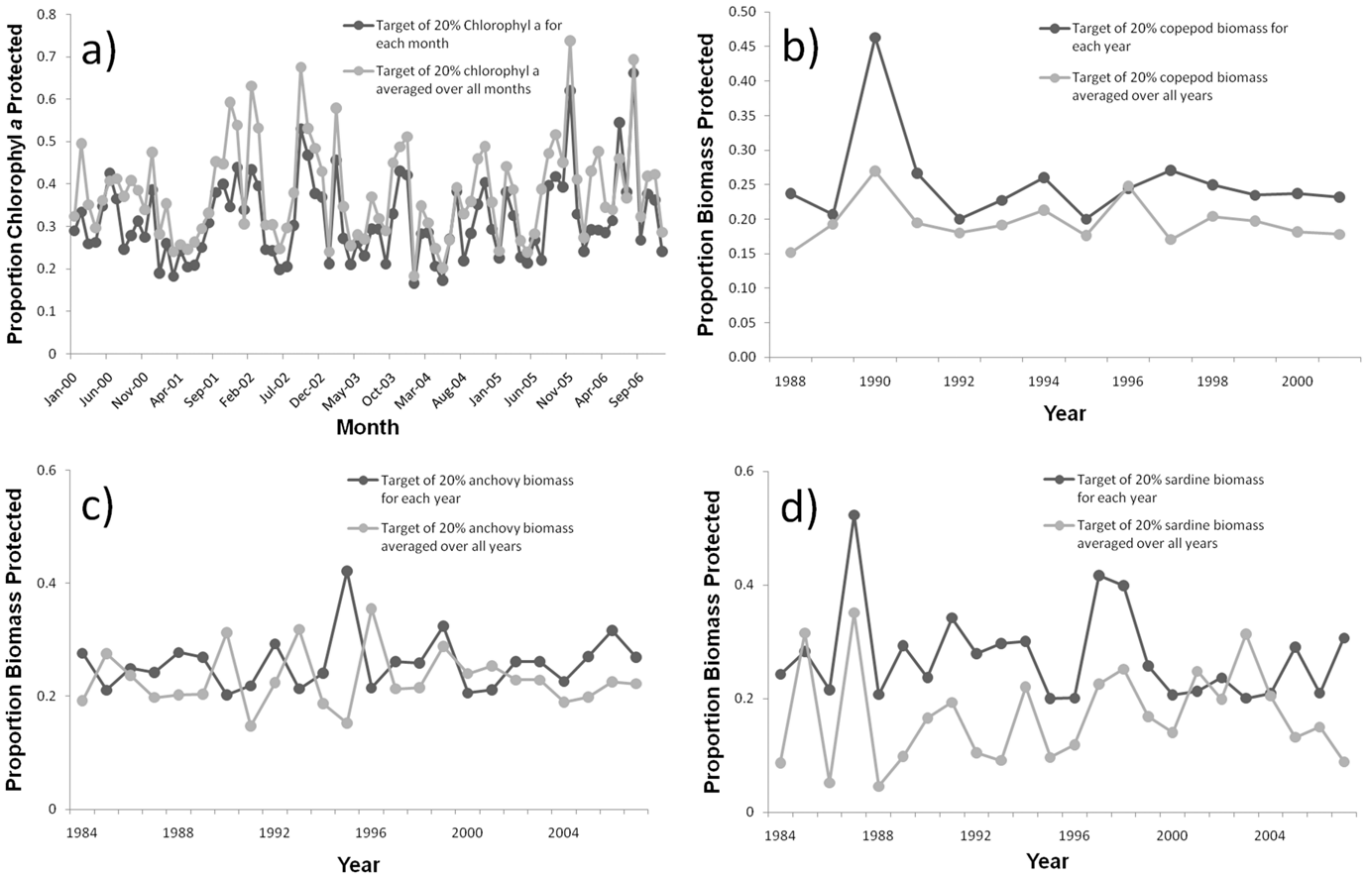
Proportion of feature protected when selections were based on data from different time periods. Dark gray lines show level of representation in best solutions when targets were set for chlorophyll *a* (monthly), copepods (yearly), anchovies (yearly) and sardines (yearly). Light gray lines show level of representation in best solutions when targets were set for values of these four features averaged over the whole periods data availability (January 2000 to December 2006 for chlorophyll a, 1998–2001 for copepod biomass, 1984–2007 for anchovy biomass, 1984–2007 for sardine biomass). (a) proportion of chlorophyll a protected, (b) proportion of copepod protected, (c) proportion of anchovies protected, and (d) proportion of sardines protected.

Comparing the best solution for these two scenarios, we found measuring the proportion protected for chlorophyll *a* resulted in fairly similar results between the two scenarios (Fig 7a). However, for copepods, anchovies and sardines, the two approaches produced quite different results (Fig 7b–d). The 20% targets for split time periods were not achieved for many periods by selections based on targets for overall averaged values. We found that time-period targets were not achieved for 9 out of 14 periods for copepods, 7 out of 24 periods for anchovies, and 16 out of 24 periods for sardines (Fig 7d).

For scenario three we experimented with boundary lengths of adjacent planning units to produce solutions with mixed compactness, we were able to develop solutions with compactness higher offshore than inshore (Fig 6c). The best solutions required a similar number of planning units (n = 4929) compared with the solution where boundary lengths were equal (n = 4764) (Fig 6a). The selection frequencies between this scenario and scenario one were correlated albeit less so than comparisons between other scenarios (Spearman's rank correlation of 0.37, p<0.0001).

**Figure 8:**
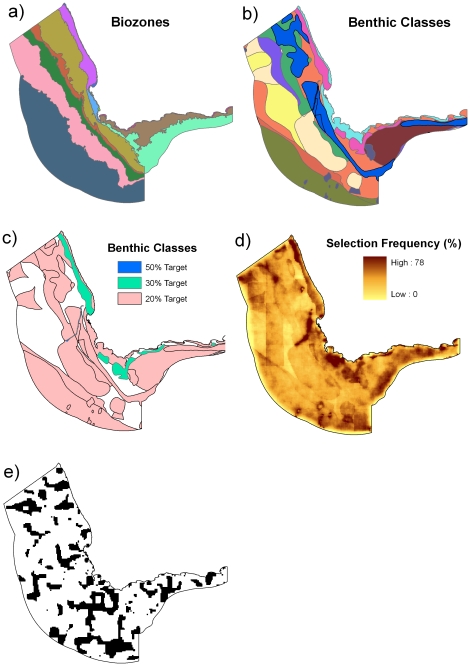
Integrated pelagic and benthic protected area design. Benthic data included two biodiversity surrogates used as a proxy for benthic biodiversity (a) biozones based on depth classes, and (b) different benthic habitat classes based on geology. Both were used as a basis for designing protected areas for benthic biodiversity. Each biozone had a target of 20% representation in protected areas. (c) different benthic habitat classes had different targets ranging from 30 to 50%. Areas were selected based on a combination of the pelagic features, biozones and benthic habitat map. (d) the selection frequency for the combined benthic and pelagic targets. (e) the most efficient solution for the combined benthic and pelagic targets.

When benthic biodiversity was included in the prioritization (Fig 8a,b), there were more areas with higher selection frequencies, indicating less spatial flexibility in the configuration of protected areas (Fig 8c). There were particularly important areas to the north west of Cape Point in a linear configuration related to a canyon. Other areas with high selection frequencies overlapped with benthic classes that had targets of 30%. The illustrative best solution (Fig 8d) contains areas selected that were scattered throughout the study region. The selection frequencies between this scenario and the main scenario were correlated (Spearman's rank correlation of 0.60, p<0.0001).

## Discussion

There is a tendency for management agencies to manage marine resources and plan management actions for individual species, separately for inshore and offshore areas, and for benthic and not pelagic habitats [58]. In this study, we successfully included spatially and temporally variable features relevant to pelagic conservation in a decision support tool (usually applied to static features) to design pelagic MPAs. Our integrated approach in this dynamic oceanographic region includes planning for multiple species and oceanographic features, both inshore and offshore areas, and considered both pelagic and benthic environments.

### Accounting for pelagic ecosystem dynamics in marine conservation planning

While a significant proportion of the study region would need to be protected in order to achieve the conservation objectives (∼20%), there was a high degree of spatial flexibility in where objectives could be achieved. Any protected area network design is likely to be most successful when it is the result of a participatory planning approach where key stakeholders are involved in decision-making about the location of conservation management [59]. A map such as the most frequently selected areas (Fig 5c) could be a good starting point for negotiation.

The extent to which large oceanic processes can be adequately protected in conservation areas depends to some extent on how the implementation of protected areas will impact stakeholders. The very large protected areas required to protected highly dynamic features might not be feasible, in which case, other forms of conservation management such as gear restrictions or market-based approaches [60] might be more appropriate. We explored spatial and temporal variability mostly using surface-measured features (e.g. eddies detected using SSH) and seafloor features (e.g. shelf break) as surrogates for water column processes. Water column processes are important drivers of productivity. However, the inclusion of vertical processes might be challenging for science and management if surface and seafloor measured features are not adequate surrogates for vertical processes.

We estimated the spatial and temporal variations in the occurrences of top predators indirectly by using time series data on chlorophyll and primary consumers. Time-series data on a monthly time scale (e.g. satellite-derived chlorophyll) are likely to capture spatio-temporal variation better than the annual time scales for primary consumers based on annual surveys. The potential advantage, however, of using data on primary consumers, despite the coarser temporal resolution, is their closer trophic relationship with top predators, although top predators also feed on mid trophic levels. For example, Gremillet *et al.* [61] tested how well primary production (based on chlorophyll and sea surface temperature) and primary consumers predicted the abundance of Cape gannets. They found that high production was a good predictor but, surprisingly, that primary consumers were not. Further work on using appropriate surrogates in the absence of data on top-predators is needed. In the Southern Ocean, Lombard *et al.* [45] used the average position of oceanic fronts as a feature in the design of pelagic MPAs around South Africa's Prince Edward Islands. There is evidence that many birds and seals forage in the vicinity of these fronts [62], [63] because of the elevated plankton and fish biomass associated with them [64]. There is also evidence that mesoscale eddies created up current of the islands are important feeding grounds for top predators [63], [65]. Our approach presented here could easily incorporate fronts that could be measured using readily available SST or Chlorophyll *a* data. Retention areas are important for fish recruitment when a species is going through passive life history stages where they cannot easily swim [20]. While these species are generally too small to be caught at this time by fisheries, the degree to which they are important feeding grounds for other species is uncertain.

To capture the spatial and temporal dynamics of this region, we set conservation targets for different time periods (e.g. multi-annual sardine abundance). We found that, to represent spatial variability in features through time, it was more effective to explicitly target this variability than to target overall average values, particularly for sardines. There was, a very minor trade-off, with only slightly more area required to capture dynamic features separated into discrete time periods than to represent overall averaged values. We did not set separate targets for different time periods for meso-scale eddies and filaments because they were more dynamic that other features, but rather identified areas where they occur most frequently. A range of alternative metrics could be used to capture dynamic features in protected areas, such as areas of low variability and/or sustained high abundance [66].

We demonstrated how artificially increasing the boundary lengths of offshore planning units resulted in solutions that were more spatially compact offshore than inshore. Such solutions might be desirable for a number of reasons: a) species tend to be more mobile offshore [20]; b) it can be difficult to enforce small offshore protected areas [15]; and c) travelling longer distances past larger protected areas from ports might be prohibitively costly for some inshore fishers.

### How would the protected area network contribute to fisheries sustainability?

The effectiveness of area closures for increasing the sustainability of fishing is uncertain, particularly for offshore areas and wide-ranging pelagic species [41]. However, there is some evidence that protected areas might benefit highly mobile species [15], [67], [68]. These benefits can be further examined with ecosystem models to test the effects of different configurations of protected areas [2], [69]. A major impediment to building spatially explicit ecosystem models has been the lack of data on dispersal parameters and seasonal migration for large pelagic species, but this is rapidly changing with the increasing number of tracking studies [70].

It is recommended that economic costs and benefits of conservation actions be incorporated into decision-support tools such as Marxan see [71] for a review. Costs are typically included as static values, whereas costs in many regions will respond dynamically to conservation decisions. We used a coarse-scale surrogate for opportunity costs and preferentially located protected areas further from ports to reduce costs to fishers. However, fishing vessels do not necessarily go to the nearest port to offload their fish, so our surrogate could be improved by including more detail on the cost-benefit relationship between the profitability of different ports and different fisheries. We recommend using more comprehensive cost data where possible. Costs of area closures based on catch and effort fisheries data, for example, could be used in further analyses. Another improvement would be the dynamic coupling between planning software and cost models, an area of current research and development [72].

Developing pelagic protected areas is one approach to conservation management in exploited pelagic regions, and might reduce the *in situ* threats from fishing. Their creation, however, is likely to impact fisheries and their management directly and indirectly and the costs and benefits of them assessed against other actions (e.g. fisheries regulation). For example, protecting an area from fishing can lead to displaced fishing effort, which could require additional management action to realize the regional benefits of protected areas [67], [73]. There are also indirect challenges associated with the creation of protected areas, in particular relating to the interpretation of biomass via traditional fisheries stock assessments, and stock monitoring [74]. Some have argued that the creation of fisheries closures will make fisheries management harder, because the underlying dynamics of fisher behavior and opportunities for fisheries-dependent data collection will be altered [74]. Fisheries assessment techniques that can overcome this problem will be needed [15], because spatial management will continue to be an important tool for conservation and fisheries management.

### Importance of pelagic protected areas for small pelagic fishes

Small pelagic fishes have an important ecological role in the Benguela ecosystem [27], [33]. We were able to use time series data on anchovies and sardines that were based on a mixture of life history stages. The fishery and ecosystem consequences of protecting only a portion of the distribution of these species are uncertain. The anchovy fishery is a recruit fishery and operates in the inshore nursery area. It is probably most important to protect spawners to improve recruitment of both species [33]. Spawners are predominantly located on the Agulhas Bank, although their location has been dynamic over time. The Agulhas Bank is also a spawning and nursery area for numerous other species and the area of highest abundance for many endemic species of fishes (e.g. Sparidae), several of which are in decline [33].

Consequences of protecting spawners are uncertain, however, as most eggs have a very low probability of survival arising from transportation off the shelf into unsuitable conditions or because of high predation risk [32], [33]. Genetic studies have shown that only a few individuals that spawn contribute to reproductive success, most likely because of patchy favourable conditions during spawning [33]. The distribution and movement of different life history stages is not well understood [75]. Additionally, sardines for example, have previously shifted their spawning location and are thought to be flexible in their selection of spawning areas [33], [76], [77].

By using time series data on anchovies and sardines we were able to locate the most predictable occurrences over time assuming that past areas will be indicative of future areas. We identified solutions that contained a proportion of total sardine abundance for each previous year. This was to try and represent the inter-year anomalies of anchovy and sardine abundance. Given that the locations of recruits and spawners can change over time, an alternative approach to using fixed locations for protected areas could be the use of a dynamic protected area system [15]. Protected area locations could be determined based on the recruitment and spawner surveys that delineated their distribution in near-real time.

### Importance of pelagic protected areas for coastal seabirds

For coastal seabirds, we identified areas that would protect their pelagic prey species from purse-seine fishers. We did this by using estimated foraging ranges of breeding seabirds, and variables such as chlorophyll as a proxy for primary production. Predictably, important areas were foraging zones around islands where the majority of colonies are located [52]. Important areas were particularly concentrated around Cape Point and in the eastern part of the study area. This analysis was based on data describing their feeding distribution during the breeding season and, whilst the distribution of these birds is likely to be different outside of the breeding season, it is during breeding times that they are most vulnerable to competition for food [50].

Although we included the most recent data on the location of breeding colonies, these localities have shifted in the past [78]. For example, three new colonies of African penguins have appeared since the 1980s [50]. If closures were to be implemented using this approach, then planners would have to decide which colonies should be included in the analysis or when to revise recommended closures as new colonies were established or old ones abandoned. While we account for within-species differences among colonies for African penguins in their foraging distances, there are likely to be other inter-colony differences for other penguins and seabirds [50], [79].

We used a baseline target of 20% of the foraging range for each seabird species. Ideally, further research is needed to decide on the most appropriate targets and configurations of protected areas and their likely influence on seabird populations e.g. [51]. For example, the energetic needs of seabirds and relationships between foraging distances and breeding success require further investigation. More information on these issues could support more specific criteria for incorporation into the analysis. Similarly, further studies that predict likely effects of closures on fishers would help to determine what management actions are feasible to protect seabirds outside, as well as inside, protected areas [80].

Applying protected areas can result in complex, uncertain, and in some situations even negative changes in seabird populations. For example, cormorants compete with the critically endangered Leach's Storm Petrel for breeding sites in South Africa [52]. Conservation management might increase the populations of cormorants but consequently reduce the availability of breeding sites for storm petrels. There are also competing and complex interactions with fishers. One hypothesis suggests possible benefits to penguins from purse-seine fishing, which disrupts shoaling defense mechanisms thereby making them more accessible to penguins [53]. Closing foraging areas to all types of fishing could be detrimental to some species. While many seabirds compete with fishers for prey, some have developed a reliance on fishery discards as a source of food [81]. Walmsley *et al.* [82] estimated that over 9000 tonnes of hake and large amounts of by-catch are discarded annually off the west and south coasts. Some bird species probably rely on these discards [50], [79], although the relationship is not well understood for some species [49]. It is likely, however, that protected areas could help increase the population viability of some seabirds in this study region.

### Importance of pelagic protected areas for pelagic bycatch species

By-catch from longline fisheries is of major conservation concern in the study region [54]. While many of these species, including those caught as by-catch, are highly mobile, they tend to aggregate in areas of high productivity such as eddies [25], [83]. Eddy activity is concentrated in the southern part of the study area and along the shelf. Protecting areas of most consistent eddy activity and those with most by-catch gives the highest probability of protected areas being effective for the species concerned. Because many of these species are wide ranging, their conservation will simultaneously depend on complementary management in other regions. Grantham *et al.* [26] investigated different approaches to fisheries closures for by-catch in the South African longline fishery and found that, because of within-species differences in where and when individuals are caught, moveable closures could minimize the impact on the longlining industry. Moveable closures could be incorporated into the approach we describe here. For longline fisheries with high bycatch, complementary and alternative types of management might be more appropriate, given the likely impact of closures to fishers. Alternative types of management include gear restrictions and other mitigation mechanisms such as excluder devices and market-based approaches such as compensatory mitigation [54], [60].

### Conclusion

Our intention here was to investigate an approach for identifying pelagic protected areas rather than provide a prescriptive conservation solution for the southern Benguela and Agulhas Bank ecosystems. Accordingly, our analysis was completed without stakeholder consultation that is critical for successful implementation of protected areas [84]. To credibly engage stakeholders and plan pelagic protected areas, we must fill the gaps in our knowledge of how spatial management might protect pelagic biodiversity. For the southern Benguela and Agulhas Bank ecosystems, more research would be beneficial on how spatial protection influences pelagic breeding seabirds, fisheries catch and bycatch species. It would also be beneficial to better understand the dynamics of displaced fishing effort as a result of spatial management and its influence on the effectiveness of spatial conservation management. Broader challenges include accounting for benthic and pelagic coupling, resolving how climate change will alter pelagic processes, and demonstrating the likely effectiveness of spatial management given the large movements of many pelagic species [15]. Our knowledge of how to best do pelagic conservation planning is in its infancy, however, some of these lessons can only be learned through the establishment of pelagic protected areas that can be used to advance our understanding of the role they have in the future sustainable management of the ocean. Despite uncertainty, planning should always proceed in the context of uncertainty, and that the burden of proof should not rest solely on those promoting conservation.

## Materials and Methods

Our study area was the southern Benguela and Agulhas Bank region within South African waters (Fig. 1), which we divided into 23,476 square planning units, most of which covered 25 km^2^, although those along land or political boundaries were smaller. The resolution of planning units was chosen due to match the scale of the input data.

### Oceanographic data

The shelf break was identified as the continental margin from maps produced by the South African Council for GeoScience (Fig 2a). The four seamounts were identified from marine chart SAN 4, Hydrographic Office, South African Navy (Fig 2a). We assume the area of influence of these structures to be approximately 10 km each side of the shelf break and a radius of 10 km around each seamount. While this was somewhat arbitrary, it was an estimate based on Hobday [85] and Campbell & Hobday [86] who found that juvenile southern bluefin tuna are often aggregated around 23 km from the shelf and within 5 km from seamounts.

We identified coastal upwelling areas using chlorophyll *a* concentrations measured from the SeaWiFS satellite for the period 1 January 2000 to 31 December 2006, composited at a temporal resolution of 8 days and spatial resolution of 0.0833°. Clouds can inhibit visible radiation, leading to lower recorded chlorophyll values or missing pixels in these images. We therefore developed monthly composite images based on the highest pixel value during a monthly period, and repeated this for each of the 72 months. We capped the highest value for any pixel at 10 mg m^−3^ [87] to remove potentially suspect values for single pixels, even though some higher legitimate values might occur in some inshore parts of the Benguela region.

Upwelling and downwelling features included offshore eddies and filaments. Upwelling features are often included in pelagic conservation planning primarily due to them being a good indicator for top predators. We also included downwelling features due to several reasons. These areas are likely to contain high biodiversity in the warmer and more stable areas outside upwelling areas [88], they are likely to contain some unique biodiversity compared to upwelling features and surrounding areas, and many downwelling features often have a deep chlorophyll maximum layer at the base of the thermocline, below the optical depth of satellites that can have a thin layer with relatively high chlorophyll [89]. We identified these using data on sea surface height for the same time period (8 days) as the analysis of the chlorophyll data. We used a gridded MSLA (Maps of Sea Level Anomaly) product produced by AVISO (based on TOPEX/Poseidon, Jason 1, ERS-1, ERS-2, Envisat and GFO) [90]. This product provides sea level anomalies relative to a 7-year mean from 1993 through 2003. Data provided a temporal resolution of 7 days and a spatial resolution of 0.33° on a Mercator grid and were corrected for all geographical errors. Upwelling (negative anomaly) and downwelling (positive anomaly) features were identified separately in each image [66]. For upwelling and downwelling features, strength and persistence are key determinants of increased primary productivity and thus aggregations of biota [91]. Anomaly height is indicative of both characteristics and, for this offshore region, we considered only anomalies ±10 cm to represent significant upwelling or downwelling features [66]. We then calculated the proportion of time a pixel had an upwelling or downwelling feature across all images.

Retention areas are important for fish recruitment and production of food for many life stages [20], [33]. We used results from a Lagrangian particle-tracking model that simulated oceanographic conditions to predict areas of retention described in [92]. It was based on an existing southern Benguela Regional Ocean Modelling System (ROMS) three dimensional hydrodynamic model [93]. The model was seeded every two weeks from 1992 to 1999 with 200,000 particles released across the south Benguela region. Retention was defined as the proportion of total particles released that remained within 50 km from where they were released 14 days previously. This proportion of particles was averaged over depth within each grid cell see [92].

To help predict areas where top predators occur we used time-series data of copepod biomass from zooplankton samples collected annually between 1988 and 2001 during spring/summer hydro-acoustic stock-assessment surveys of pelagic fishes. Copepods were collected from the upper 200 m using a vertically-hauled paired Bongo net system (0.57-m diameter, 200-µm mesh) preserved in 5% buffered formalin. For details on analysis and biomass calculations see Huggett *et al.* [94]. For each year, we developed a predictive layer of copepod biomass distribution by applying an inverse distance weighting extrapolation in ArcGIS version 9.2 (ESRI) across all survey points within a radius of 50 km from any data point.

### Species data

We were unable to access data for all pelagic fisheries species targeted in the region. We used distribution maps of relative abundance for round herring, snoek, chokka squid, chub mackerel, horse mackerel, big eye tuna, yellow fin tuna, and albacore tuna. These data were based on several sources including commercial fisheries and research surveys and were previously mapped on a 10′ by 10′ cell grid see [46]. Density estimates of anchovies and sardines were determined from biannual acoustic surveys between 1984 and 2007 [95], which entailed a recruitment survey during winter and a spawner biomass survey during summer. These surveys cover the entire distribution of anchovies and sardines recruits and adults and are conducted along a series of randomly-spaced parallel transects perpendicular to the coast. Distribution maps of anchovies and sardines for each survey were produced from densities, derived from hydro-acoustic surveys and estimated along sections of transects typically less than 10 nautical miles long. Linear kriging algorithms were used to interpolate densities between transects using the software Surfer [96].

We used Kemper *et al.* [52] to identify the breeding distributions of coastal seabirds that are likely to be threatened by fisheries. While their distribution during non-breeding times might be different than during breeding, of interest here is their distribution during breeding periods when they guard eggs or chicks and are limited to feeding relatively close to their nests. We followed Kemper *et al.* [52] to determine their distribution based on the location of nesting sites and estimated foraging distances. Maximum foraging distances were 40 km for African penguins (*Spheniscus demersus*), except at Boulders where it was 20 km, 10 km for bank cormorants (*Phalacrocorax lucidus*), 40 km for Cape cormorants (*Phalacrocorax capensis*), 100 km for Cape gannets (*Morus capensis*), and 2 km for roseate terns (*Sterna dougallii*). Other coastal seabirds were not included in the analysis because they are currently not known to be threatened by fishing.

We mapped the distribution of seven of the most frequently caught by-catch species in the South African pelagic longline fishery. These include three seabirds (black-browed albatross, shy albatross, and white-chinned petrel), two turtles (leatherback and loggerhead), and two sharks (short-finned mako and blue). Distribution data were collected by independent fishery observers aboard vessels in the South African pelagic longline fishery from 1998 to 2005 (South African Marine and Coastal Management unpublished data). Data were aggregated into one-degree grid cells because of limited accuracy in reported catch position owing to the length of longlines. Bycatch rates were divided by the observed fishing effort and were averaged over all years. For an overview of the spatial and temporal dynamics of these species, see Grantham *et al.* [26].

### Marxan analysis

We used the conservation planning software Marxan to identify multiple, efficient configurations of planning units that achieve a set of representation targets for conservation features while minimizing the cost to stakeholders [43]. These solutions can be indicative for locations of new protected areas. For each conservation feature, a quantitative target was set, indicating the minimum representation of that feature required within protected areas. This included each species, as well as both fixed and flexible processes. Some species and processes were separated into multiple unique features to reflect substantial intra-annual changes, such that individual targets were set for: each monthly composite map of coastal upwelling (thus n = 84 maps); annual maps of copepod biomass (n = 14 maps); and annual density distributions of anchovies and sardines (for both n = 24 maps). A baseline target of 20% was applied for all conservation features based on a general recommendation that 20–50% of marine areas should be included in protected areas [40], [66]. We expect that other targets would be explored when stakeholders are involved in conservation planning. For features with data on distribution, we used area as a basis for calculating the target. For abundance or density data, we calculated the target by summing all values across planning units and calculating 20% of the total.

We used a surrogate for the cost of conservation to the fishing industry, so that we could find protected area solutions that minimized the overall burden on the industry. We used distance to port as our cost surrogate, with the closure of areas closer to port having higher costs to the fishery. Fuel and wages are important costs to fishers, so excluding fishing from areas closer to port would increase their costs. Given our aim here is to demonstrate a technique, we did not attempt to deal comprehensively with costs. In a real conservation planning exercise, however, we recommend that more detailed data on catch and effort and associated cost-benefit ratios should be used where possible, along with any other data on human-uses that might be affected by conservation management [97].

By adjusting a Marxan parameter called the “boundary length modifier” (BLM), the level of spatial compactness of a solution can be controlled because it places more or less emphasis on reducing the summed boundary length of selected areas [98]. By experimenting with a range of BLM values and visually inspecting the results, we identified a modifier that ensured solutions were adequately compact. Marxan uses a simulated annealing algorithm to identify a range of possible protected area solutions [43]. This algorithm has a randomization component and therefore potentially results in a different solution during each run. Marxan was run 1000 times (each with 1000000 iterations). Each run produces a different solution. Two results were extracted: the ‘best solution’ and ‘selection frequency’. The ‘best solution’ is the set of planning units that best achieves targets for conservation features, minimizes cost and minimizes boundary length. The selection frequency is the number of times a particular planning unit was selected across all 1000 runs and is used as an indication of conservation importance. A value of 500, for example, indicates that a planning unit was selected in 50% of the Marxan runs.

We applied three more scenarios to the one described above. For each comparison we compared the number of planning units in the best solution. We also measured the correlation in selection frequencies using a spearman rank correlation. Higher values indicated a more similar spatial pattern in selection frequencies.

For scenario two, we compared our approach to a scenario that used, instead of data for separate “slices” of time (Table 1), data based on average values over the time period considered. For this “average” scenario, we used the same features and targets, with the exception of several features. For coastal upwelling we used the average over all months (n = 1 vs 84 separate monthly averages). Similarly, copepod biomass and density distributions of anchovies and sardines were averaged over all years of data. In addition to comparing the number of planning units in the best solution and the correlation between selection frequencies, we measured and compared the proportion protected at each time period retrospectively. For example for coastal upwelling we measured how much was protected (based on the best solution) for at each 84 separate monthly averages.

For scenario three, we experimented with BLM values to produce solutions where compactness was higher offshore than inshore. This might be useful because species are generally wider ranging offshore than inshore (e.g. birds dispersing from a colony to offshore feeding grounds). To achieve this range of compactness, we multiplied by 10 all boundary lengths of planning units 20 km beyond the shelf.

For scenario four we included a scenario that combined benthic and pelagic protected area design. We used two data sources: viz. surface sediments (hereafter “habitats”) described in Dingle *et al.* [99] and Lombard *et al.* [100], and ‘biozones’, which were based on dividing the region into depth classes and stratifying these by bioregion. Bioregions were an updated version described in Lombard *et al.* [100]. The current version was based on new depth classes and bioregions that were revised, with new biological data (K. Sink pers. comm.). The different classes of habitat and biozone were used as general surrogates for benthic biodiversity [45]. In a real conservation planning exercise, we expect that more comprehensive data on benthic biodiversity would be sought.

Targets for habitats were based on Driver *et al.* [38], who used a target of 20% of their total area, with a few exceptions. For authigenic sediments, terrigenous muds and currently untrawlable grounds on the Agulhas Bank, they used a target of 30%, and for canyons, they used 50%. Currently untrawlable grounds contain a mixture of rocky and soft-bottom communities. These soft-bottom communities are heavily trawled elsewhere, but could be trawled in the future with new bobbin trawling gear. Some habitats did not have targets because their value as surrogates was questionable (P. Ramsay and A. Connell, pers. comm.). All biozones had a target of 20%.
